# Determination of muscle shape deformations of the tibialis anterior during dynamic contractions using 3D ultrasound

**DOI:** 10.3389/fbioe.2024.1388907

**Published:** 2024-06-05

**Authors:** Annika S. Sahrmann, Lukas Vosse, Tobias Siebert, Geoffrey G. Handsfield, Oliver Röhrle

**Affiliations:** ^1^ Institute for Modelling and Simulation of Biomechanical Systems, University of Stuttgart, Stuttgart, Germany; ^2^ Stuttgart Center for Simulation Science, University of Stuttgart, Stuttgart, Germany; ^3^ Institute of Sport and Movement Science, University of Stuttgart, Stuttgart, Germany; ^4^ Auckland Bioengineering Institute, University of Auckland, Auckland, New Zealand

**Keywords:** 3D ultrasound, image processing, dynamic movement, muscle deformation, contraction

## Abstract

**Purpose:**

In this paper, we introduce a novel method for determining 3D deformations of the human tibialis anterior (TA) muscle during dynamic movements using 3D ultrasound.

**Materials and Methods:**

An existing automated 3D ultrasound system is used for data acquisition, which consists of three moveable axes, along which the probe can move. While the subjects perform continuous plantar- and dorsiflexion movements in two different controlled velocities, the ultrasound probe sweeps cyclically from the ankle to the knee along the anterior shin. The ankle joint angle can be determined using reflective motion capture markers. Since we considered the movement direction of the foot, i.e., active or passive TA, four conditions occur: slow active, slow passive, fast active, fast passive. By employing an algorithm which defines ankle joint angle intervals, i.e., intervals of range of motion (ROM), 3D images of the volumes during movement can be reconstructed.

**Results:**

We found constant muscle volumes between different muscle lengths, i.e., ROM intervals. The results show an increase in mean cross-sectional area (CSA) for TA muscle shortening. Furthermore, a shift in maximum CSA towards the proximal side of the muscle could be observed for muscle shortening. We found significantly different maximum CSA values between the fast active and all other conditions, which might be caused by higher muscle activation due to the faster velocity.

**Conclusion:**

In summary, we present a method for determining muscle volume deformation during dynamic contraction using ultrasound, which will enable future empirical studies and 3D computational models of skeletal muscles.

## 1 Introduction

Muscles are the motors of human movement. During contraction, muscle deformation occurs as a result of changes in muscle length, changes in internal muscle architecture (particularly pennation angle and fascicle length), and constraints on muscle deformation by surrounding tissues and external forces (Lieber and Fridén, 2000; Reinhardt et al., 2016; Wick et al., 2018).

2D ultrasound is a widely-used and clinically established tool for investigating skeletal muscle architecture (Arampatzis et al., 2006; Ryan et al., 2019). In a systematic review, Hooren et al. (2020) showed the feasibility of 2D ultrasound for investigating fascicle length and pennation angle during dynamic contraction. However, with such examinations, 3D information is missing which may provide relevant information on the muscle’s contractile behavior. A commonly used method to examine 3D muscle architecture and muscle volume relies on a combination of conventional magnetic resonance imaging (MRI) and diffusion tensor imaging (DTI) (van Donkelaar et al., 1999; Heemskerk et al., 2010; Hiepe et al., 2013). Compared to such methods, ultrasound is less expensive, enables faster acquisitions and is portable.

Thus, there have been recent efforts to generate 3D images from ultrasound data. In this regard, muscle volume is a useful morphological parameter to infer age, training level and growth processes of subjects (Albracht et al., 2008; Hanson et al., 2009; Siebert et al., 2017). In addition, the anatomical cross-sectional area (CSA) and its distribution on the muscle belly can provide information about the specific muscle shape and muscle adaptations (Albracht et al., 2008).

3D freehand ultrasound is an approach for acquiring muscle volumes from ultrasound imaging where the position of the ultrasound probe is recorded by, e.g., optical retroreflective motion capture markers or a magnetic sensor. The operator uses the equipped probe to scan along the longitudinal muscle axis, such that the image position and orientation for each cross-sectional 2D image is known. As a result, a stack of 2D cross-sectional images of the muscle is collected, from which a 3D volume can be reconstructed by applying a series of coordinate transformations (Barber et al., 2009; Rana and Wakeling, 2011; Raiteri et al., 2016; Weide et al., 2017). Furthermore, the reconstructed volumes obtained from 3D freehand ultrasound measurements can be used to determine in-plane fascicle lengths or pennation angles (Kurihara et al., 2005; Weide et al., 2017).

Previous studies which investigated volumetric muscle data using 3D ultrasound mainly focused on isometric contractions or resting states (Raiteri et al., 2016; Weide et al., 2017; Cenni et al., 2018). Isometric conditions are static and account only for a small part of movements in everyday life. There is a need for acquisition techniques that capture the dynamic contractile behavior of muscles that investigate 3D muscle deformation in dynamic conditions. Such a technique could record dynamic muscle shape changes during contraction which would capture essential information that could be used as inputs for the development and validation of 3D computational models of muscles (Röhrle et al., 2016; Seydewitz et al., 2019).

This study presents a novel approach for determining 3D deformation of the tibialis anterior (TA) muscle during dynamic movement. The approach represents an extension of an automated 3D ultrasound system for acquisition of muscle volumes in static conditions (Sahrmann et al., 2024; Sahrmann et al., 2024), and a sophisticated implementation of the methods employed in Sahrmann et al. (2022). By using motion capture for determination of the ankle joint angle and encoder data for recording the ultrasound probe position and orientation, each 2D ultrasound image contains a corresponding probe position/orientation and an ankle joint angle. Reconstructing 3D ultrasound images while the foot is moving is possible by defining ankle joint angle intervals during repeated plantarflexion movements. In this study, we determine muscle volume and the anatomical CSA from the acquired information, i.e., the 2D images, as well as position and ankle joint angle information.

## 2 Materials and methods

This section describes the experimental methods for obtaining 3D ultrasound images in dynamic conditions, i.e., while the subject actively moves the foot under controlled conditions. The algorithm for reconstructing 3D volumes of the TA during motion is presented, which groups ankle joint angles into intervals of 1°.

### 2.1 Experimental setup

We used an automated 3D ultrasound system, with an Aixplorer MACH30 (Supersonic Imagine, Aix-en-Provence, France) and a linear probe (SuperLinear SL18-5), for acquisition of 3D ultrasound data, as further described in Sahrmann et al. (2024). The system consists of three axes along which the probe is moved and a built-in force control mechanism to ensure consistent tissue deformation. Simultaneously, eight infrared cameras (VICON, Oxford, United Kingdom) recorded positional data from reflective markers at a frame rate of 100 Hz. The system contains three movement directions, which allow positioning of the ultrasound probe in the 3D space. The first movement direction corresponds to the circular path of the two semicircles. The second movement axis corresponds to the horizontal axis between the two semicircles, which allows the movement along the shank (longitudinal muscle axis). The third movement axis corresponds to the vertical movement of the probe, perpendicular to the longitudinal axis. This vertical axis is realized as a direct linear motor and contains an included force control, such that the probe presses on the skin with the same force along the scanning trajectory. With this, there is no loss of skin contact due to the curved shape of the leg or inconsistent tissue deformation due to changes in the exerted contact force.

We collected the 2D ultrasound images with an HD frame grabber (USB3HDCAP, Star-Tech.com Ltd.) at a frame rate of 30 Hz. For synchronizing the encoder data of the axes of the system, the ultrasound images recorded with the frame grabber and the VICON motion capture markers, the automated 3D ultrasound system at first sent a 5 V signal to the VICON system once a movement at the axes is started or stopped. This signal serves as an external trigger for VICON to start or stop a measurement. Simultaneously, VICON sends out a User Datagram Protocol (UDP) message containing relevant information if a recording starts or stops. A custom-written LabVIEW (version 2021.0) script receives the UDP message as a trigger for recording the image data. For computation of the ankle joint angle, reflective markers were placed at the following locations: lateral and medial epicondyle, lateral and medial malleolus, first and fifth metatarsal. We placed each subject’s lower limb on a hard cushion. The positioning allowed movement of the foot and visibility of the reflective markers.

In total, 3D ultrasound images of the TA of the right leg were obtained from five subjects (2 male, three female). The experimental procedures involving human subjects described in this paper were approved by the University of Stuttgart’s Committee on Responsibility in Research (number: Az. 21-011). All subjects provided written informed consent. The anthropometric characteristics of the subjects are: age 27.8 ± 3.1 years, height 177.2 ± 7.9 cm, weight 68 ± 10.9 kg, body mass index 21.52 $\pm 1.93\frac{kg}{m^{2}}$ .

As a first part of the study, participants were told to actively move their foot to two different positions: (1) plantarflexion and (2) neutral foot position, which is defined as the neutral standing position. One scan of each position was conducted per subject with an average scan time of 15–20 s. The probe movement velocity on the horizontal axis was set to 20 $\frac{mm}{s}$ for the static and dynamic trials. With a recording frame rate of 30 Hz, there are approximately 15 images collected per cm the average distance between two sequential collected 2D images is 0.67 mm.

For the second part of the study, the subject performed a cyclical movement consisting of a plantarflexion (neutral to plantarflexed position) and a dorsiflexion (from plantarflexed to neutral position) until a defined stop signal is given. Average joint angles for plantarflexion and the neutral position were 157° ± 6° and 113° ± 9°, respectively.

We used a metronome for visual and audio feedback for the subject to control their foot velocity. Moving velocity was set on the metronome to 45 and 100 beats per minute, which is referred to as *slow* and *fast*, respectively. This means that the foot was in plantarflexion on every second beat, and in the neutral position on each beat in between.

**FIGURE 1:**
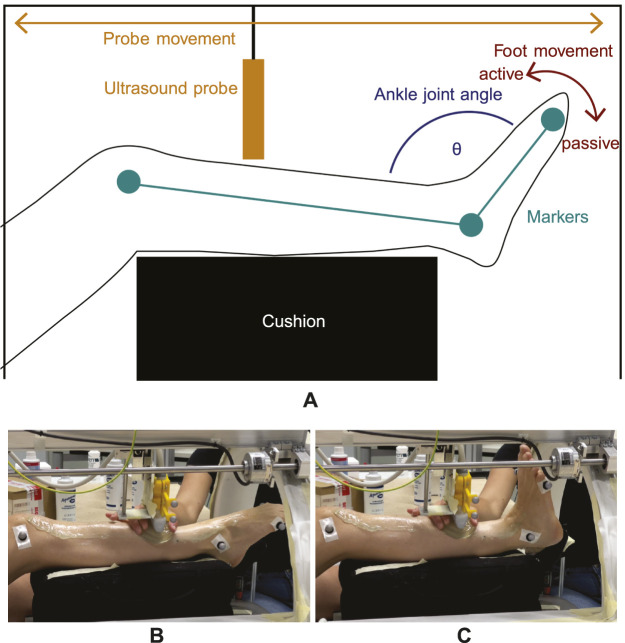
Experimental setup for dynamic imaging. **(A)** The lower leg is elevated and equipped with reflective markers (green) from which the ankle joint angle *θ* (blue) can be computed. While the foot moves periodically from plantarflexion to neutral position (red arrow) and vice-versa, the transducer moves back and forth (orange arrow) from the distal to the proximal end of the TA. As the dorsiflexion movement direction is generated by TA contraction, it is defined as an *active* movement whereas opposite plantarflexion is defined as a *passive* movement. **(B,C)** show images of the setup with one subject during dynamic imaging at two different ankle joint angle positions. Here, the gel pad is moved alongside to ensure sufficient image quality.

Simultaneously, the automated 3D ultrasound system moved the transducer periodically from the proximal to the distal end of the TA and vice-versa (along the horizontal axis, Figure 1).

The principle of the data acquisition method is illustrated in Figure 1. Since the repetitive foot movement is a monotonous task, it can be demanding in terms of coordination and concentration. Therefore, each subject’s trial for a given velocity (which totaled 9 min) was split into 4 separate smaller trials of 2 min and 15 s each, in order to ensure a constant concentration level for the subjects during the measurements. To avoid loss of skin contact, a gel pad (Aquaflex, Parker Laboratories, Fairfield, United States of America) and ultrasound gel were used (see Figures 1B, C).

### 2.2 Angle intervals

After recording, encoder positions and motion capture data were sampled to the ultrasound frame rate. Four trials of one velocity were stitched to obtain a sufficient number of 2D images within one ankle joint angle interval for reconstruction. The ankle joint angle *θ* was computed as the angle between the vector from lateral knee epicondyle to lateral malleolus and the vector from lateral malleolus to fifth metatarsal (see Figure 1A). Ultrasound measurements for a given ankle angle were binned into intervals of 1°, which means *θ* − 0.5° ≤ *θ* < *θ* + 0.5°. Furthermore, it was determined if the slope on the ankle angle curve was negative or positive. This was done to define the direction of the foot movement, i.e., if it is a dorsiflexion (negative slope) or plantarflexion (positive slope) movement. Since 3D ultrasound images of the TA were acquired, the dorsiflexion induced by TA contraction is referred to as *active* movement direction and the opposite plantarflexion movement direction is referred to as *passive* in the following. All frames and encoder positions belonging to one specific angle interval and movement direction were extracted.

**FIGURE 2:**
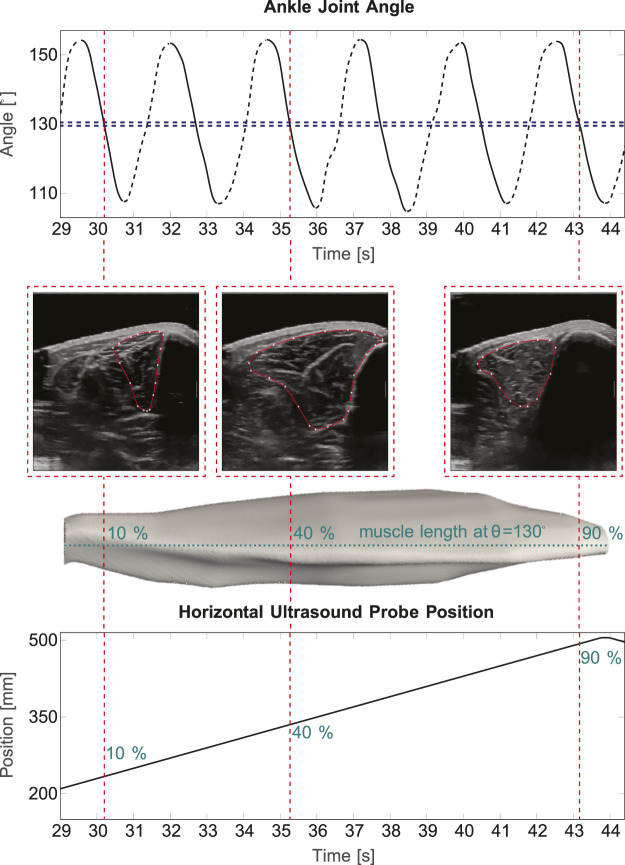
3D ultrasound in cyclic movements. The top part of the figure shows a 15 s sequence of measured ankle joint angle during a cyclic *slow* velocity trail. Here, it is distinguished between *active* (solid lines) and *passive* (dashed lines) movement. A 1° interval at 130° ankle joint angle is illustrated exemplarily in blue, the red lines show three example image frames, which are extracted. Below, the corresponding 2D B-Mode ultrasound images and the corresponding position on the muscle are shown. The bottom part displays the ultrasound probe’s position along the horizontal axis of the device.

Figure 2 illustrates a cutout (of approximately 15 s) of one example trial with *slow* velocity. Three extracted frames at the same ankle joint angle *θ* (130°), yet from different positions along the TA (10%, 40% and 90% of the muscle length), are shown exemplarily for *active* dorsiflexion.

If the distance between two images within one interval was smaller than 0.8 mm, the average of those images and corresponding position values was taken. The image stack and the corresponding encoder positions were exported to Matlab (R2020a, MathWorks, MA, United States of America) if they met the following criteria: 1) a minimum of 40 images are within the image stack of the interval with one movement position and 2) the maximum distance between two images within the stack is less than 25 mm.

A Stradwin (v6.02, University of Cambridge, United Kingdom) file was generated from the Matlab file containing all 2D images and position information for a given *θ* interval. Stradwin is a freely available software tool developed by the Machine Intelligence Laboratory at the Cambridge University’s Engineering Department. The program was developed mainly for data acquisition and visualization of 3D freehand ultrasound applications. Stradwin requires two different input files: a binary image file (*.sxi*) containing the collected images and a text file (*.sw*) comprising pixel scaling information for each image and associated position and orientation data. In the program, the 2D images are automatically positioned according to the position and orientation data in the *sw* file.

Since there are four different conditions (*slow active*, *slow passive*, *fast active*, *fast passive*), the maximum and minimum available *θ* meeting the inclusion criteria was determined for each condition. For each subject, range of motion (ROM) was defined as the range from the larger value of the smallest available *θ* value for *active* and *passive* movement to the smaller value of the largest available *θ* value for *active* and *passive* movement. For each of the four conditions, the volume was reconstructed in 10% intervals of ROM. This means, that there are 11 reconstructions (0%–100% of ROM) for each subject and condition, so a total of 44 data sets (which describe the 3D muscle shape) per subject. The ROM intervals correspond to the muscle elongation caused by extension of the foot for plantarflexion, i.e., the 0% of ROM means the shortest TA muscle length and 100% of ROM refers to the longest one.

### 2.3 3D reconstruction and image segmentation

We used Stradwin for 3D reconstruction of the TA. The segmentation was done by manually outlining the muscle on 15–20 slices at approximately equidistant frames along the muscle’s longitudinal axis. The selection of 15-20 segmented slices leads to a slightly larger distance between images in comparison to a study of Raiteri et al. (2016), investigating the TA in isometric conditions using 3D freehand ultrasound. Here, the TA cross-section was segmented in 5–15 mm intervals. Both the muscle volume and length and the fascicle orientations and lengths were investigated, whereas our study primarily focuses on the assessment of muscle volume, length and anatomical cross-sectional area (CSA). From the manually outlined slices, Stradwin interpolates a surface through the segmented image slices and creates a 3D volume (Treece et al., 1999; Treece et al., 2000). Surface creation settings in Stradwin were set to *low resolution* and *high smoothing* strength.

We used a weighted principal component analysis (PCA), as proposed in the study of Raiteri et al. (2016), to initially rotate the geometry into the coordinate system of its principal component axes. Volume was defined as the sum of the segmented voxels multiplied by their resolution in each dimension. Length is the euclidean distance from the centroids of the first and last segmented slice, e.g., the most proximal and most distal point of the TA. For each segmentation slice, the CSA is computed by the sum of pixels multiplied by the pixel size.

### 2.4 Ellipsoid prediction

An ellipsoid can be considered a highly simplified shape of a muscle which has been shown to allow reproduction of muscle shapes and gearing ratios (Siebert et al., 2012). For later comparison with experimental TA data, the equation for the volume of an ellipsoid was used for computing its CSA (Eq. (1)):

$$V=\frac{4}{3}\pi abc.$$ (1)

Here, *a*, *b*, *c* are the lengths of the semi-axes of the ellipsoid. The parameter *c* is defined as the longitudinal semi-axis of the ellipsoid (Eq. (2)),

$$c=\frac{1}{2}L_{\mathrm{Muscle}},$$ (2)

where *L*_*Muscle*_ is the muscle length at the corresponding *θ* interval. The maximum CSA of an ellipsoid can be defined as (Eq. (3)):

$$CSA_{\mathrm{Ellipsoid}}=\pi ab.$$ (3)

Muscle volume was assumed to stay constant (Baskin and Paolini, 1967; Böl et al., 2013), while *c* was considered to elongate as the muscle would be lengthened by moving the foot from neutral position to plantarflexion. Using the experimentally determined CSA values, *CSA*_*ellipsoid*_ can be determined as (Eq. (4)):

$$CSA_{\mathrm{Ellipsoid}}=\frac{3}{4}\frac{V_{\mathrm{muscle}}}{c}.$$ (4)

### 2.5 Statistical analysis

Shapiro-Wilk tests were used for testing the data for normal distribution. For comparison between groups, one way repeated analyses of variance (ANOVAs) were used for normally distributed data and Friedman tests were applied for non-normally distributed data. Analyses of covariance were employed for comparison between regression lines. The level of significance, *α*, was set to 0.05.

## 3 Results

**FIGURE 3:**
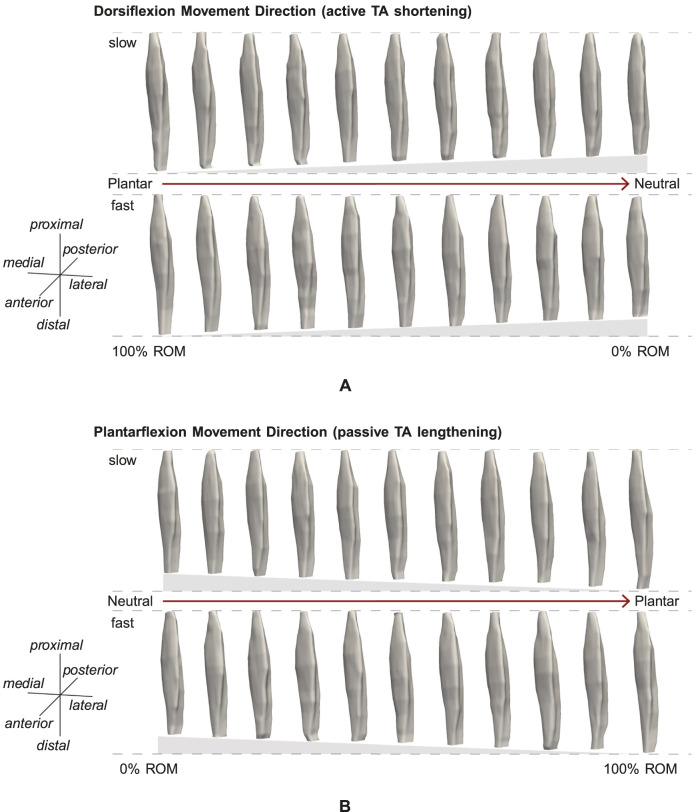
Example reconstruction for 10% intervals of ROM for **(A)** *active* and **(B)** *passive* movement with *slow* and *fast* velocity. **(A)** Muscle shortening towards the neutral position can be observed. **(B)** Muscle lengthening and stretching towards the plantarflexion position are visible.

Figures 3A, B show examples of TA volume reconstructions for one representative subject for all 11 intervals of ROM, for *active* TA shortening and *passive* TA lengthening, respectively. As expected, muscle length decreases during *active* TA contraction with a decrease in the ankle joint angle, i.e., a decrease in ROM (100% ROM to 0% ROM, see Figure 3A). Due to TA shortening the muscle gets thicker.

### 3.1 Muscle volume and length

The mean computed muscle volume of the five subjects for the static trials was 89.0 ± 28.1 cm^3^. For these trials, muscle volumes did not differ significantly between neutral position and plantarflexion for each subject. For the dynamic trials, the determined mean muscle volume was 89.2 ± 26.4 cm^3^. Muscle volume did not differ significantly between the different ROM intervals. Also, no significant differences between the muscle volumes obtained from dynamic and static trials could be observed. Averaged over all subjects, the maximum differences of normalized muscle volume between different ROM intervals for dynamic trials were in a 2% range. The mean change in muscle length from neutral position to plantarflexion was 20.0 ± 3.7 mm (9.6% increase) for static trials. For the dynamic trials, we found an overall length change of 22.5 ± 8.5 mm (11.2% increase) from neutral to plantarflexion. We further computed the change in muscle length for the two movement directions separately. For the *active* condition, the muscle length decrease was 23.0 ± 5.6 mm (11.5%) and for the *passive* condition, the muscle length increase was 22.0 ± 10.9 mm (10.8%).

### 3.2 Cross-sectional area

The shortest muscle length, corresponding to 0% of ROM (Figure 3), was defined as 100% of the reference muscle length *L*_0_. Muscle lengths of other ROM intervals were computed with regard to *L*_0_. Thus, by increasing the ROM intervals to 100% of ROM, the maximum muscle length is 111.2% of *L*_0_, corresponding to a mean stretch of 11.2%.

**FIGURE 4:**
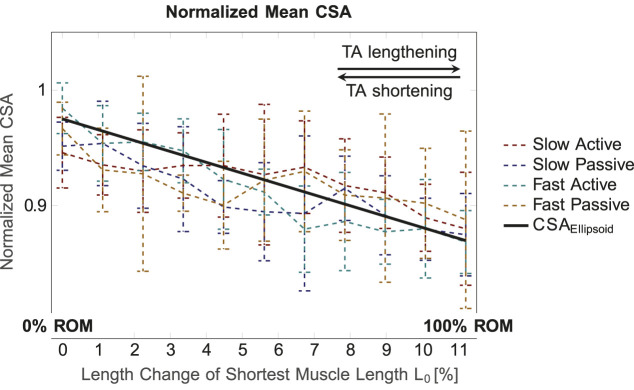
Mean normalized CSA (for the whole muscle) for the four conditions and different muscle lengths. The normalized volume value for the ellipsoid, which is defined as constant for all muscle lengths, was set to 1.3. In general, the normalized mean CSA decreases for increasing muscle length.

Figure 4 illustrates the mean normalized CSA values for the whole muscle for the four conditions (*slow active*, *slow passive*, *fast active*, *fast passive*), and the predicted ellipsoid cross sectional area *CSA*_*Ellipsoid*_. Here, *CSA*_*Ellipsoid*_ shows a straight line with a negative slope, i.e., a decrease in CSA for elongation of the ellipsoid. The experimentally determined CSA shows a similar trend for all conditions. Significant differences in mean CSA between different muscle lengths, i.e., different ROM intervals, were found for *slow active* (*p* = 0.004), *slow passive* (p < 0.001) and *fast active* (p < 0.001), and no significant differences for *fast passive* (*p* = 0.111). Mean CSA was significantly different between *slow active* and *fast active* (*p* = 0.003) and *fast active* and *fast passive* (*p* = 0.001).

**FIGURE 5:**
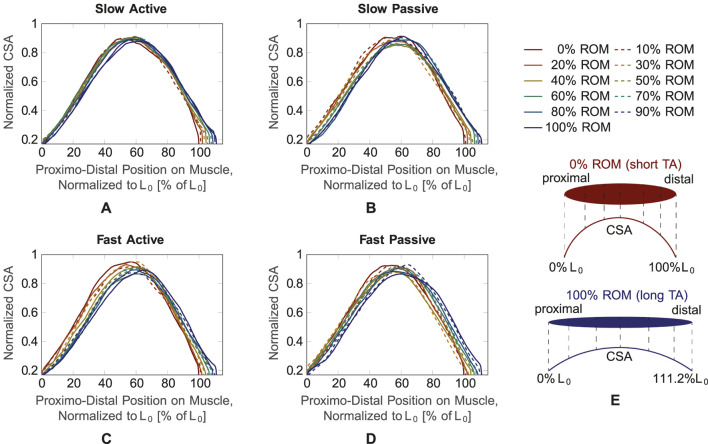
CSA over muscle length normalized to the shortest muscle length *L*_0_ for the four conditions. Different muscle lengths corresponding to different ROM values (0%–100% ROM) are plotted with different line types and colors. **(A)** *Slow active*, **(B)** *slow passive*, **(C)** *fast active*, **(D)** *fast passive*. As schematically shown in **(E)**, 0% ROM and 100% ROM refer to the shortest and longest TA length, respectively. The muscle CSA (exemplary broken vertical lines in **(E)** is plotted from the proximal to the distal end of the muscle, which corresponds to 111.2% *L*_0_ for the longest muscle.

Figure 5 illustrates the mean normalized CSA of all subjects for the four different conditions (Figures 5A–D), plotted over the shortest muscle length *L*_0_ for the dynamic trials. The curves are color-coded according to *L*_0_, i.e., from red (minimum muscle length, corresponding to 0% ROM) to blue (maximum muscle length, corresponding to 100% ROM). For better visualization, they are illustrated with alternating solid and dashed lines. For all four conditions, the curves show an increase in CSA in the middle region of the muscle, while they narrow towards the proximal and distal ends. This can be explained by the spindle-like shape of the muscle, i.e., the muscle belly is located in the middle part of the muscle and narrows towards the tendons at the proximal and distal part of the muscle (Figure 5E). It can be observed that the maximum value of the CSA is shifted in the left direction on the *x*-axis, i.e., towards the proximal side, for decreasing muscle length, which is especially visible for the *fast active* condition (Figure 5C). Thus, for the more proximal part of the muscle, the red curve (0% ROM) is above the blue curve (100% ROM). In the distal part of the muscle, the red curve is below the blue one. During *fast active* contractions (Figure 5C) there is a noticeable increase in the maximum CSA (blue line, representing 100% ROM to red line, representing 0% ROM) as the muscle shortens.

Due to slight fluctuations in the curves, which may be caused by movement and evaluation artifacts, assessments of the three other conditions (*slow active*, *slow passive*, *fast passive*) are difficult. To achieve a better understanding of the changes in maximum CSA and its position on the longitudinal axis of the muscle, we smoothed the curves by fitting them with a second degree polynomial function.

**FIGURE 6:**
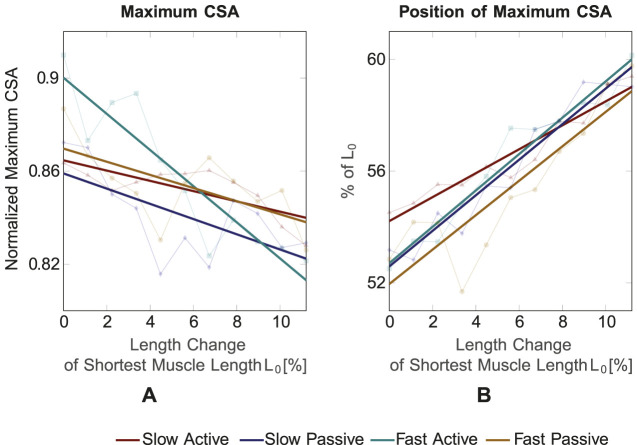
**(A)** Maximum CSA value and **(B)** position of maximum CSA for the four conditions and different muscle lengths. The maximum CSA decreases for increasing muscle length and its position shifts towards the distal part for increasing muscle length.

For testing of correlation between the curves and the polynomial fit, a Pearson correlation was applied and a mean correlation coefficient *R* = 0.981 (ranging from 0.967 to 0.989) was found. Thus, the polynomial fit can be considered a reliable approximation. The resulting maximum CSA values and their corresponding positions on the muscle’s longitudinal axis for the four conditions (pale colored lines) are shown in Figures 6A, B, respectively. In general, for muscle shortening, the CSA seems to increase by approximately 5% (Figure 6A) and its position seems to shift from ∼60% of *L*_0_ to ∼55% of *L*_0_, i.e., in proximal direction on the longitudinal muscle axis (Figure 6B). To confirm these statements statistically and to examine the differences between the four conditions, an analysis of covariance, which makes use of linear regressions, was employed (colored thick lines in Figure 6). Based on the analysis of covariance, we can conclude that in all four conditions a decrease in the maximum CSA for stretching the muscle can be observed.

Maximum CSA is significantly different between *slow active* and *fast active* (*p* = 0.005), *slow passive* and *fast active* (*p* = 0.028) and *fast active* and *fast passive* (*p* = 0.0014). The position of the maximum CSA shifts to the proximal part for shorter muscle lengths for all four conditions. The position of the maximum CSA does not significantly differ between the four conditions.

## 4 Discussion

In this paper, we introduced a novel approach for obtaining 3D muscle volumes of the TA during movement. In general, the TA muscle consists mainly of type I muscle fibers (Henriksson-Larsén et al., 1983; Jakobsson et al., 1990). The contraction velocity and forces (∼0N) required to perform the prescribed movement are notably lower than the muscle’s capacity. Participants characterized the movement’s difficulty as minimal, with no indication of perceived exhaustion. Consequently, muscular fatigue is not deemed a limiting factor. In both static and dynamic conditions, muscle volumes stayed constant while muscle lengthening was observed due to an increase of ROM. This is consistent with previous literature (Fry et al., 2003; Barber et al., 2009) and may indicate a low error of the proposed method for determining muscle shape and volume in dynamic conditions. So far, 3D freehand ultrasound imaging studies on skeletal muscles mainly focused on isometric contractions, i.e., static conditions, for obtaining 3D volumes (Barber et al., 2009; Weide et al., 2017; Cenni et al., 2018). With the proposed method, 3D reconstructions of the TA during periodic movements are possible. This may provide entirely new means to investigate the muscle’s dynamic contraction behavior.

### 4.1 Comparison with a simple ellipsoidal muscle model

When the ankle joint angle decreases, the TA muscle shortens and the maximum CSA shifts towards the proximal origin of the muscle. Since the muscle volume stays constant, it has to redistribute along the shorter length of the muscle. The comparison of the CSA prediction by an ellipsoidal muscle model with experimentally obtained data shows a similar trend in decrease of CSA for increase in muscle length (Figure 4). This demonstrates that the proposed method can serve as a suitable tool for determining 3D muscle deformations during dynamic contractions.

Although an ellipsoid is a highly simplified shape of a skeletal muscle, it has been shown to be an appropriate geometric body for predicting muscle shape and contraction behavior (Siebert et al., 2012). However, the suitability for the ellipsoid as a representative muscle shape (Aubel and Thalmann, 2004) may vary between different muscles. This applies particularly to non-spindle-shaped muscles with more complex muscle geometries, such as the back and shoulder muscles (Stark et al., 2012; Tous et al., 2023). Thus, the proposed method enables the generation of more sophisticated input geometries for modeling skeletal muscle, compared to a simplified shape, such as an ellipsoid.

### 4.2 Comparison of 2D and 3D ultrasound

Franchi et al. (2017) found positive correlations between muscle thickness measured from 2D ultrasound images and anatomical CSA obtained from MRI. Investigating the TA by using 2D ultrasound, Maganaris and Baltzopoulos (1999) and Reeves and Narici (2003) did not find significant changes in TA muscle thickness at different ankle joint angles. Their results applied to resting conditions and maximum voluntary contraction (MVC). In the present study, we found an increase of the mean CSA (Figure 4) and maximum CSA (Figure 6) with a decrease of the ankle joint angle in *passive* and *active* conditions. In terms of the relationship between the anatomical CSA and muscle thickness (Franchi et al., 2017), this would not be consistent with the findings of Maganaris and Baltzopoulos (1999) and Reeves and Narici (2003). Hu et al. (2019) found, however, significant differences between muscle thickness for dorsiflexion and plantarflexion and Choi et al. (2019) observed increases in muscle thickness during contraction.

Such differences in findings may be explained by the different methods used in the studies or differences in TA architecture of the examined subject groups. The TA has a complex bipennate architecture and exhibits sexual dimorphisms in humans (Martin-Rodriguez et al., 2023). Furthermore, the TA presents morphological asymmetries between its superficial and deep unipennate regions. For example, the superficial and deep TA regions differ in thickness and pennation angle (Martin-Rodriguez et al., 2023). Depending on their architecture, muscles and muscle compartments can deform differently in their width and thickness when shortened (Eng et al., 2018). Observed muscle deformations via 2D ultrasound may therefore depend on the positioning and orientation of the ultrasound probe. Thus, the existing differences in findings on 2D thickness indicate the need of a method for obtaining 3D volumes of muscles revealing deformation patterns during dynamic contractions, of which our proposed method is capable.

### 4.3 Impact of movement condition on CSA

Significant differences in mean CSA were observed for different ROM intervals for the *slow active*, *slow passive* and *fast active* conditions but not for the *fast passive* one (see Section 3.2 and Figure 4). Increasing the movement velocity during contractions against equal loads (in our case the inertia of the foot) requires higher muscle activations. Thus, it can be expected that the *fast passive* TA movements required a higher activation of the antagonistic plantar flexors (i.e., the gastrocnemius muscles and the soleus muscle) compared to *slow passive* TA movements. In previous studies on the human lateral gastrocnemius, a gradual increase in muscle activation from 10% to 100% resulted in different muscle thicknesses (Kelp et al., 2023). This is due to activation and force-dependent changes in the internal muscle architecture, especially the pennation angle. At higher contraction speeds there is greater muscle fiber rotation, i.e., change in pennation angle (Azizi et al., 2008; Eng et al., 2018). This can also lead to movement velocity dependent differences in CSA. Since the lower leg rested on the plantar flexors in our experimental setup (Figure 1), their higher *active* deformation at increased cycle frequencies (100 beats per minute) led to a stronger vertical movement of the lower leg and the ultrasound transducer. Thus, artifacts in the ultrasound TA images may occur. Consequently, such vertical movement may induce the larger standard deviations in the computed CSA observed, as in Figure 4 (yellow broken line). Hence, the scatter of the data for the *fast passive* condition is larger, which is probably why statistically significant deviations do not occur.

We found significantly different maximum CSA values between the *fast active* and all other conditions. The observed differences are explainable and to be expected because the muscle shape differs between *active* and *passive* muscles with the same length (Böl et al., 2013). The linear regression of the maximum CSA curve (Figure 6A) shows the steepest slope for the *fast active* condition, where the highest TA muscle activation can be expected. This indicates a greater bulging of the muscle for the *fast active* condition. The findings are in agreement with those of Raiteri et al. (2016), who measured CSA changes of the TA during isometric contractions with different activation levels using 3D freehand ultrasound. For low activation levels in a range of 5%–10% MVC, the change in CSA compared to a resting state was in a range of 2%–4%, which is relatively small. Larger increases in CSA were observed for 25% and 50% MVC.

The increase in CSA for higher velocities can be related to the variable contraction behavior of a muscle and the architectural gearing ratio (AGR) (Brainerd and Azizi, 2005). The AGR is defined as the relation of the muscle shortening velocity to the fiber shortening velocity. When a muscle contracts, variable shape changes of the muscle can occur (Eng et al., 2018), i.e., the thickness can increase, decrease or stay constant. This is caused by the architectural changes in the muscle during dynamic contraction, as muscle fibers can both shorten and rotate. The combination of shortening and rotation enables changes in the pennation. Thus, changes in pennation angle allow the muscle belly to shorten faster than the fibers, which refers to an AGR greater than one. In this study, the increase in CSA for faster velocities may therefore be caused by a higher AGR.

### 4.4 Limitations

One limitation of the proposed method is that the leg of the subject was not fixed during scanning. Therefore, it was not possible to enable a fully controlled movement of the leg. This means that side movements of the foot during *active* or *passive* movement were possible. Further, some participants felt a moderate discomfort and needed to shift the leg’s position between trials, even though the movement was relatively small, since the subjects stayed in the lying position and did not stand up in between. Thus, the stitched trials might not always be recorded at the exact same leg position. Moreover, the movement velocity of the foot was based on the metronome. This does not ensure that all movements were exerted with the exact same velocity. Therefore, in order to ensure more controlled conditions in terms of movement direction and velocity, future studies should include a fixture of the leg which enables only plantarflexion and dorsiflexion movements. The fixation of the leg and the kinematic control of a reproducible cyclic joint movement may be realized by using an isokinetic measurement system, such as an ISOMED (Holzer et al., 2023). Through the possibility of more controlled measurements with less movement artifacts, the deformation gradient of the muscle may be computed. This means that 3D deformation changes can be determined between different muscle lengths during dynamic movements. By reducing the effects of movement and fixing the leg, it is possible to examine not only the external deformation of the muscle, but also its internal architecture, for which the method described in Sahrmann et al. (2024) can be used. Furthermore, with our algorithm, 3D images were reconstructed if the maximum distance between two images did not exceed 25 mm. In our study, the main focus was to present a method for obtaining features such as muscle volume, length and CSA, where this distance was sufficient for segmentation. However, this distance could limit reconstructions in terms of fascicle features such as fascicle length or pennation angle. For also determining such fascicle characteristics, the maximum distance between two sequential images would need to be reduced. Therefore, with a decrease of the maximum distance and the previously mentioned solutions for avoding artifacts due to unwanted subject movements, fascicle parameters such as fascicle length, pennation angle and the physiological cross-sectional area may be determined in future studies.

Another limitation is that no EMG was applied, therefore the activation level of the muscle was not measured. Arampatzis et al. (2006) stated that the TA is active during maximal plantarflexion efforts. In our case, however, the movement is a free movement without any resistance, therefore we can assume overall small activation levels of the dorsi- and plantarflexors. With regard to the plantar flexion movement, we can assume that we are at a low activation level of the plantarflexors and therefore also have minimal activation of the TA. Due to this very low activation of the TA, we assume that the TA has no influence here, thus we chose the terminologies *active* and *passive* based on these assumptions. Due to the low level of TA activation, differences in CSA between *active* and *passive*, and *slow* and *fast* velocities were comparably small. Consequently, it would be beneficial to include a force resistance and measurement of muscle activity, to also study effects of the CSA during higher muscle contraction levels. Thus, future studies should contain a mechanism, such that the foot moves against a controlled resistance, e.g., a dynamometer, and EMG electrodes. This would also enable measurements where to foot is moved passively.

Furthermore, since the aim of this work is to present a novel method to obtain and reconstruct 3D ultrasound images of the TA during dynamic movements, we employed the method on five human subjects. This number of subjects might be a limiting factor in this study, as a larger population can increase the level of confidence in our results and conclusions. In addition, due to the small number of subjects we did not examine our data for gender-specific differences that may affect musculature and height. Thus, in future studies a larger number of subjects should be investigated such that also gender-specific data can be examined.

## 5 Conclusion

In summary, we have proposed a method for determining muscle volume deformation during dynamic contraction. Constant muscle volumes during contraction indicate the suitability of the method for determination of TA shape changes during dynamic movements. Moreover, we determined realistic changes in 3D muscle shape, such as the proximal shift in maximum CSA. Thus, an improved understanding of muscle contraction behavior, especially during dynamic movements, can be achieved with the proposed method. Since the acquisition of 3D deformation of skeletal muscle during dynamic movement is highly restricted with the current existing imaging methods, this study contributes significantly to the research area.

Data on changes in muscle shape during contraction are needed for the validation of 3D muscle models (Seydewitz et al., 2019). Therefore, the proposed method may help to enhance input data for existing computational models and to answer new research questions. In addition, new insights into the muscle geometry in dynamic conditions may help to establish new means to investigate skeletal muscles in healthy and pathological conditions. As such, further extensions may enable the development of new therapy approaches.

## Data Availability

The raw data supporting the conclusion of this article will be made available by the authors, without undue reservation.

## References

[B1] Albracht K. Arampatzis A. Baltzopoulos V. (2008). Assessment of muscle volume and physiological cross-sectional area of the human triceps surae muscle *in vivo* . J. Biomechanics 41, 2211–2218. 10.1016/j.jbiomech.2008.04.020 18555257

[B2] Arampatzis A. Karamanidis K. Stafilidis S. Morey-Klapsing G. DeMonte G. Brüggemann G.-P. (2006). Effect of different ankle- and knee-joint positions on gastrocnemius medialis fascicle length and EMG activity during isometric plantar flexion. J. Biomechanics 39, 1891–1902. 10.1016/j.jbiomech.2005.05.010 15993886

[B3] Aubel A. Thalmann D. (2004). MuscleBuilder: a modeling tool for human anatomy. J. Comput. Sci. Technol. 19, 585–595. 10.1007/bf02945584

[B4] Azizi E. Brainerd E. L. Roberts T. J. (2008). Variable gearing in pennate muscles. Proc. Natl. Acad. Sci. 105, 1745–1750. 10.1073/pnas.0709212105 18230734 PMC2234215

[B5] Barber L. Barrett R. Lichtwark G. (2009). Validation of a freehand 3d ultrasound system for morphological measures of the medial gastrocnemius muscle. J. Biomechanics 42, 1313–1319. 10.1016/j.jbiomech.2009.03.005 19375081

[B6] Baskin R. Paolini P. (1967). Volume change and pressure development in muscle during contraction. Am. J. Physiology-Legacy Content 213, 1025–1030. 10.1152/ajplegacy.1967.213.4.1025 6051170

[B7] Böl M. Leichsenring K. Weichert C. Sturmat M. Schenk P. Blickhan R. (2013). Three-dimensional surface geometries of the rabbit soleus muscle during contraction: input for biomechanical modelling and its validation. Biomechanics Model. Mechanobiol. 12, 1205–1220. 10.1007/s10237-013-0476-1 23417261

[B8] Brainerd E. L. Azizi E. (2005). Muscle fiber angle, segment bulging and architectural gear ratio in segmented musculature. J. Exp. Biol. 208, 3249–3261. 10.1242/jeb.01770 16109887

[B9] Cenni F. Schless S.-H. Bar-On L. Aertbeliën E. Bruyninckx H. Hanssen B. (2018). Reliability of a clinical 3d freehand ultrasound technique: analyses on healthy and pathological muscles. Comput. Methods Programs Biomed. 156, 97–103. 10.1016/j.cmpb.2017.12.023 29428080

[B10] Choi M.-S. Shin J.-H. Park H.-K. Lee W.-H. (2019). Reliability and validity of rehabilitative ultrasound images obtained using a hands-free fixed probe in measuring the muscle structures of the tibialis anterior and the gastrocnemius. Phys. Ther. Rehabilitation Sci. 8, 194–201. 10.14474/ptrs.2019.8.4.194

[B11] Eng C. M. Azizi E. Roberts T. J. (2018). Structural determinants of muscle gearing during dynamic contractions. Integr. Comp. Biol. 58, 207–218. 10.1093/icb/icy054 29889236 PMC6104701

[B12] Franchi M. V. Longo S. Mallinson J. Quinlan J. I. Taylor T. Greenhaff P. L. (2017). Muscle thickness correlates to muscle cross-sectional area in the assessment of strength training-induced hypertrophy. Scand. J. Med. Sci. Sports 28, 846–853. 10.1111/sms.12961 28805932 PMC5873262

[B13] Fry N. Childs C. Eve L. Gough M. Robinson R. Shortland A. (2003). Accurate measurement of muscle belly length in the motion analysis laboratory: potential for the assessment of contracture. Gait Posture 17, 119–124. 10.1016/s0966-6362(02)00059-0 12633771

[B14] Hanson E. D. Srivatsan S. R. Agrawal S. Menon K. S. Delmonico M. J. Wang M. Q. (2009). Effects of strength training on physical function: influence of power, strength, and body composition. J. Strength Cond. Res. 23, 2627–2637. 10.1519/jsc.0b013e3181b2297b 19910811 PMC2966873

[B15] Heemskerk A. M. Sinha T. K. Wilson K. J. Ding Z. Damon B. M. (2010). Repeatability of DTI-based skeletal muscle fiber tracking. NMR Biomed. 23, 294–303. 10.1002/nbm.1463 20099372 PMC4416059

[B16] Henriksson-Larsén K. B. Lexell J. Sjöström M. (1983). Distribution of different fibre types in human skeletal muscles. i. method for the preparation and analysis of cross-sections of whole tibialis anterior. Histochem. J. 15, 167–178doi. 10.1007/BF01042285 6343306

[B17] Hiepe P. Herrmann K.-H. Güllmar D. Ros C. Siebert T. Blickhan R. (2013). Fast low-angle shot diffusion tensor imaging with stimulated echo encoding in the muscle of rabbit shank. NMR Biomed. 27, 146–157. 10.1002/nbm.3046 24151092

[B18] Holzer D. Millard M. Hahn D. Siebert T. Schwirtz A. Seiberl W. (2023). Tendon compliance and preload must be considered when determining the *in vivo* force–velocity relationship from the torque–angular velocity relation. Sci. Rep. 13, 6588. 10.1038/s41598-023-33643-9 37085664 PMC10121672

[B19] Hooren B. V. Teratsias P. Hodson-Tole E. F. (2020). Ultrasound imaging to assess skeletal muscle architecture during movements: a systematic review of methods, reliability, and challenges. J. Appl. Physiology 128, 978–999. 10.1152/japplphysiol.00835.2019 32163334

[B20] Hu C. Hu H. Mai X. Lo W. L. A. Li L. (2019). Correlation between muscle structures and electrical properties of the tibialis anterior in subacute stroke survivors: a pilot study. Front. Neurosci. 13, 1270. 10.3389/fnins.2019.01270 31849584 PMC6902003

[B21] Jakobsson F. Borg K. Edström L. (1990). Fibre-type composition, structure and cytoskeletal protein location of fibres in anterior tibial muscle. Comparison between young adults and physically active aged humans. Acta Neuropathol. 80, 459–468doi. 10.1007/BF00294604 2251902

[B22] Kelp N. Y. Clemente C. J. Tucker K. Hug F. Pinel S. Dick T. J. M. (2023). Influence of internal muscle properties on muscle shape change and gearing in the human gastrocnemii. J. Appl. Physiology 134, 1520–1529. 10.1152/japplphysiol.00080.2023 37167262

[B23] Kurihara T. Oda T. Chino K. Kanehisa H. Fukunaga T. Kawakami Y. (2005). Use of three-dimensional ultrasonography for the analysis of the fascicle length of human gastrocnemius muscle during contractions. Int. J. Sport Health Sci. 3, 226–234. 10.5432/ijshs.3.226

[B24] Lieber R. L. Fridén J. (2000). Functional and clinical significance of skeletal muscle architecture. Muscle & Nerve 23, 1647–1666. 10.1002/1097-4598(200011)23:11<1647::aid-mus1>3.3.co;2-d 11054744

[B25] Maganaris C. N. Baltzopoulos V. (1999). Predictability of *in vivo* changes in pennation angle of human tibialis anterior muscle from rest to maximum isometric dorsiflexion. Eur. J. Appl. Physiology 79, 294–297. 10.1007/s004210050510 10048637

[B26] Martin-Rodriguez S. Gonzalez-Henriquez J. J. Galvan-Alvarez V. Cruz-Ramírez S. Calbet J. A. Sanchis-Moysi J. (2023). Architectural anatomy of the human tibialis anterior presents morphological asymmetries between superficial and deep unipennate regions. J. Anat. 243, 664–673. 10.1111/joa.13864 36999195 PMC10485583

[B27] Raiteri B. J. Cresswell A. G. Lichtwark G. A. (2016). Three-dimensional geometrical changes of the human tibialis anterior muscle and its central aponeurosis measured with three-dimensional ultrasound during isometric contractions. PeerJ 4, e2260. 10.7717/peerj.2260 27547566 PMC4974924

[B28] Rana M. Wakeling J. M. (2011). *In-vivo* determination of 3d muscle architecture of human muscle using free hand ultrasound. J. Biomechanics 44, 2129–2135. 10.1016/j.jbiomech.2011.05.026 21664617

[B29] Reeves N. D. Narici M. V. (2003). Behavior of human muscle fascicles during shortening and lengthening contractions *in vivo* . J. Appl. Physiology 95, 1090–1096. 10.1152/japplphysiol.01046.2002 12740314

[B30] Reinhardt L. Siebert T. Leichsenring K. Blickhan R. Böl M. (2016). Intermuscular pressure between synergistic muscles correlates with muscle force. J. Exp. Biol. 219, 2311–2319. 10.1242/jeb.135566 27489217

[B31] Röhrle O. Sprenger M. Schmitt S. (2016). A two-muscle, continuum-mechanical forward simulation of the upper limb. Biomechanics Model. Mechanobiol. 16, 743–762. 10.1007/s10237-016-0850-x 27837360

[B32] Ryan D. S. Stutzig N. Siebert T. Wakeling J. M. (2019). Passive and dynamic muscle architecture during transverse loading for gastrocnemius medialis in man. J. Biomechanics 86, 160–166. 10.1016/j.jbiomech.2019.01.054 30792071

[B33] Sahrmann A. S. Gizzi L. Zanker A. Handsfield G. G. Röhrle O. (2022). “Dynamic 3d ultrasound imaging of the tibialis anterior muscle,” in 2022 44th Annual International Conference of the IEEE Engineering in Medicine& Biology Society (EMBC), China, 11-15 July 2022 (IEEE). 10.1109/embc48229.2022.9871352 36086433

[B34] Sahrmann A. S. Handsfield G. G. Gizzi L. Gerlach J. Verl A. Besier T. F. (2024). A system for reproducible 3d ultrasound measurements of skeletal muscles. IEEE Trans. Biomed. Eng., 1–12doi. 10.1109/tbme.2024.3359854 38285583

[B35] Sahrmann A. S. Vosse L. Siebert T. 3D ultrasound-based determination of skeletal muscle fascicle orientations. Biomech Model Mechanobiol (2024). 10.1007/s10237-024-01837-3 PMC11341646 38530501

[B36] Seydewitz R. Siebert T. Böl M. (2019). On a three-dimensional constitutive model for history effects in skeletal muscles. Biomechanics Model. Mechanobiol. 18, 1665–1681. 10.1007/s10237-019-01167-9 31102082

[B37] Siebert T. Günther M. Blickhan R. (2012). A 3d-geometric model for the deformation of a transversally loaded muscle. J. Theor. Biol. 298, 116–121. 10.1016/j.jtbi.2012.01.009 22251888

[B38] Siebert T. Tomalka A. Stutzig N. Leichsenring K. Böl M. (2017). Changes in three-dimensional muscle structure of rabbit gastrocnemius, flexor digitorum longus, and tibialis anterior during growth. J. Mech. Behav. Biomed. Mater. 74, 507–519. 10.1016/j.jmbbm.2017.07.045 28778781

[B39] Stark H. Fröber R. Schilling N. (2012). Intramuscular architecture of the autochthonous back muscles in humans. J. Anat. 222, 214–222. 10.1111/joa.12005 23121477 PMC3632226

[B40] Tous C. Jodoin A. Pontré B. Grabs D. Begon M. Bureau N. J. (2023). Characterizing the myoarchitecture of the supraspinatus and infraspinatus muscles with mri using diffusion tensor imaging. J. Magnetic Reson. Imaging 59, 851–862. 10.1002/jmri.28840 37316960

[B41] Treece G. Prager R. Gee A. Berman L. (2000). Surface interpolation from sparse cross sections using region correspondence. IEEE Trans. Med. Imaging 19, 1106–1114. 10.1109/42.896787 11204848

[B42] Treece G. M. Prager R. W. Gee A. H. Berman L. (1999). Fast surface and volume estimation from non-parallel cross-sections, for freehand three-dimensional ultrasound. Med. Image Anal. 3, 141–173. 10.1016/s1361-8415(99)80004-8 10711996

[B43] van Donkelaar C. C. Kretzers L. J. G. Bovendeerd P. H. M. Lataster L. M. A. Nicolay K. Janssen J. D. (1999). Diffusion tensor imaging in biomechanical studies of skeletal muscle function. J. Anat. 194, 79–88. 10.1046/j.1469-7580.1999.19410079.x 10227669 PMC1467896

[B44] Weide G. van der Zwaard S. Huijing P. A. Jaspers R. T. Harlaar J. (2017). 3d ultrasound imaging: fast and cost-effective morphometry of musculoskeletal tissue. J. Vis. Exp., 55943. 10.3791/55943 29286445 PMC5755508

[B45] Wick C. Böl M. Müller F. Blickhan R. Siebert T. (2018). Packing of muscles in the rabbit shank influences three-dimensional architecture of m. soleus. J. Mech. Behav. Biomed. Mater. 83, 20–27. 10.1016/j.jmbbm.2018.04.006 29656240

